# Semicarbazide Accumulation, Distribution and Chemical Forms in Scallop (*Chlamys farreri*) after Seawater Exposure

**DOI:** 10.3390/ani11061500

**Published:** 2021-05-21

**Authors:** Lihong Xing, Weihong Sun, Xiaojie Sun, Jixing Peng, Zhaoxin Li, Panpan Zhu, Xuying Zheng

**Affiliations:** 1 Key Laboratory of Testing and Evaluation for Aquatic Product Safety and Quality, Ministry of Agriculture and Rural Affairs, Qingdao 266071, China; xinglh@ysfri.ac.cn (L.X.); sunxj@ysfri.ac.cn (X.S.); pengjx@ysfri.ac.cn (J.P.); Z3068707271@163.com (P.Z.); z_xy0101@163.com (X.Z.); 2 Yellow Sea Fisheries Research Institute, Chinese Academy of Fishery Sciences, Qingdao 266071, China; 3 Pilot National Laboratory for Marine Science and Technology, Qingdao 266237, China; 4 Collaborative Innovation Center of Seafood Deep Processing, Dalian Polytechnic University, Dalian 116034, China

**Keywords:** semicarbazide, accumulation, chemical forms, distribution, scallop

## Abstract

**Simple Summary:**

Semicarbazide is considered the characteristic metabolite of nitrofurazone and it is often used as a marker to monitor the illegal use of nitrofurazone in foods. Recent studies have indicated that semicarbazide pollution can be introduced in many ways and this compound is a newly recognized pollutant type in the environment that accumulates in aquatic organisms throughout the food chain. Scallops are the third most consumed shellfish in China. We therefore studied the accumulation, chemical forms, and distribution of semicarbazide in scallop tissues. Semicarbazide added to tank seawater resulted in its accumulation in both free and tissue-bound forms and the levels varied according to tissue and were present in all tissues examined. The levels were highest in viscera and the lowest in muscle. The levels of semicarbazide in the environment and in cultured shellfish should be monitored to ensure food quality and safety and human health.

**Abstract:**

Semicarbazide is a newly recognized marine pollutant and has the potential to threaten marine shellfish, the ecological equilibrium and human health. In this study, we examined the accumulation, distribution, and chemical forms of semicarbazide in scallop tissues after exposure to 10, 100, and 1000 μg/L for 30 d at 10 °C. We found a positive correlation between semicarbazide residues in the scallops and the exposure concentration (*p* < 0.01). Semicarbazide existed primarily in free form in all tissues while bound semicarbazide ranged from 12.1 to 32.7% and was tissue-dependent. The time for semicarbazide to reach steady-state enrichment was 25 days and the highest levels were found in the disgestive gland, followed by gills while levels in gonads and mantle were similar and were lowest in adductor muscle. The bioconcentration factor (BCF) of semicarbazide at low exposure concentrations was higher than that at high exposure concentrations. These results indicated that the scallop can uptake semicarbazide from seawater and this affects the quality and safety of these types of products when used as a food source.

## 1. Introduction

Semicarbazide is a characteristic metabolite of nitrofurazone and often used as a marker to monitor the illegal use of nitrofurazone in foods. Nitrofurazone is a nitrofuran drug used as an antimicrobial in aquaculture and livestock farming to treat *Escherichia coli*, *Staphylococcus saprophyticus*, *Enterococcus faecalis*, *Citrobacter* spp., as well as *Vibrio cholera* [1]. Nitrofurans are potentially hazardous substances associated with carcinogenic, teratogenic, and mutagenic effects [2,3,4,5]. Because of food safety and health issues, the use of nitrofurans in food and animal production was banned by the European Commission 95/1442/EC [6], USA (Federal Register 2002) [7], China (Regulation of the ministry of Agriculture of China 2002) [8] and Korea (Food code 2004).

Recent studies have indicated that the metabolic degradation of nitrofurazone was not the only source of semicarbazide residues in aquatic products [9]. Semicarbazide is frequently used as a photochromic dye for the preparation of thermal recording paper and as an intermediate for drugs such as adrenobazone and prednisone [10,11]. Semicarbazide is also released as a byproduct of azodicarbonamide used as a food additive or when packaging is decomposed at a high temperature [12,13,14]. Semicarbazide could also be detected in food by treating with hypochlorite [15,16,17] and exists naturally in some crustacean products and red seaweed and carrageenan [15,18,19].

Toxicological studies have shown that semicarbazide inhibits semicarbazide sensitive amine oxidases, lysyl oxidase, and glutamic acid decarboxylase (GAD) [20,21,22]. Semicarbazide also had a remarkable anti-estrogenic effect on female zebrafish (*Danio rerio*) that resulted in significant decreases in mRNA from the *vtg-1*, *ERα*, and *ERβ* genes [23]. Semicarbazide disturbs the reproductive system of male zebrafish through the GABAergic system and exerted anti-androgenic effects in male flounders by altering the HPG axis, kiss/gpr54 system and GABA synthesis [24,25]. Additionally, semicarbazide can significantly downregulate expression of *kiss*2, *gpr*54, and the GABA synthesis enzyme *gad*67 and exerted anti-estrogenic effects in female flounders [26]. Semicarbazide also adversely affected the structure of the respiratory tree, intestines and muscles of the sea cucumber *Apostichopus japonicas* [27].

There are various sources of semicarbazide and when discharged into the environment, it becomes a pollutant and accumulates in aquatic organisms. Seawater and sediments have varied levels of this compound and residual semicarbazide can be detected in marine organisms and its presence was first reported in the aquatic environment in 2010 [28]. That study investigated the pollution status of semicarbazide in seawater, sediment, and shellfish near the Chaohe River estuary and semicarbazide levels were 0.18–70.6 μg/L, 0.26–18.9 μg/kg, and 0.82–6.46 μg/kg, respectively. Semicarbazide in seawater and shellfish in western Laizhou Bay were 10^−11^ and 10^−10^ kg/L, respectively [29]. The semicarbazide levels in Jincheng and Sishili bays for seawater and shellfish and sediments were 0.011–0.093 μg/L and 0–0.75 μg/kg [30], respectively. These results indicated that shellfish and other organisms can uptake semicarbazide from seawater. This affects the quality and safety of these food products and can harm human health, as well as the food chain.

Nitrofurazone possesses a very short in vivo half-time and is metabolized rapidly after it enters the animal body, but its metabolite semicarbazide exists in tissue-bound form and is persistent for several weeks or longer [19,31,32,33,34]. The bound semicarbazide cannot be completely degraded by common cooking methods such as frying, grilling, roasting, or microwaving [35] while the free semicarbazide can be removed by washing [36,37]. There are few reports about the forms of semicarbazide that are present in polluted environments and the scope of its presence in shellfish products is unclear.

The deterioration of the environment including coastal waters and sediments by semicarbazide pollution affects the safety of shellfish products. Scallop is one of the most important economic species in marine culture in China for a long time. According to FAO (2020), scallops accounted for 11.0% of total mollusc production in 2018 [38]. In China, the production of scallop was 1.83 million tons, ranking third in seawater shellfish production [39]. *Chlamys farreri* is one of the most important marine economic cultured scallop in North China. We examined whether scallops accumulate semicarbazide in either a bound or free form. We examined the existing form, distribution, and bioaccumulation characteristics of semicarbazide in *Chlamys farreri* in the current study.

## 2. Materials and Methods

### 2.1. Chemicals and Reagents

Methanol, ammonium acetate, ethyl acetate, dimethyl sulfoxide, 2-nitrobenzaldehyde were of high-performance liquid chromatography (HPLC) grade and purchased from Merck Corporation (Darmstadt, Germany). Dipotassium hydrogen phosphate and hydrochloric acid were of superior pure grade and purchased from Sinopharm Chemical Reagent (Shanghai, China). Semicarbazide HCl and semicarbazide HCl-^13^C,^15^N_2_ analytical standards were obtained from Dr.Ehrenstorfer GmbH (Augsburg, Germany). Water was purified in a Milli-Q system (Millipore, Burlington, MA, USA). Semicarbazide crude drug (99% purity) was obtained from Aladdin (Shanghai, China).

### 2.2. Exposure Experiment

#### 2.2.1. Scallops and Temporary Culture

Healthy scallops (with complete mantles and gills, adductor quickly responding to external stimulus) were obtained from a local farm in Qingdao and kept in a flow-through water tank for two weeks at 10 ± 1 °C with a light: dark cycle of 14 h:10 h and were fed daily. Scallop weights were 48.8 ± 5.1 g and they averaged 80 × 74.8 × 20 cm. During the experiment, the air was supplied continuously for 24 h by bubbling air through airy stones. The dissolved oxygen (DO) for the control group and experimental group was set as 7.5 ± 0.2 mg/L, with pH of 8.0 and 33‰ salinity. Neither semicarbazide nor nitrofurazone were found in the adductor muscle, mantle, disgestive gland, gills, or gonads of control scallops tested before starting the experiments.

#### 2.2.2. Medicated Bath

The scallops were randomly divided into control and treatment groups before experiments. The control group lacked semicarbazide and the treatment group was divided into low, medium and high concentration groups at 10, 100, and 1000 μg/L, respectively. Each group has three parallels, and the tanks were 70 × 50 × 25 cm in size. During the experiment, the study was performed with semi-static system, and the seawater was replaced every day. Semicarbazide crude drug was dissolved in distilled water and the stock solutions were prepared at a concentration of 10 mg/mL used for no longer than 2 weeks. The stock solutions were considered to be stable based on previous studies [24]. Daily stock aliquots were prepared by diluting this stock to produce final concentrations of 10, 100, and 1000 ng/mL. The stock solutions were stored in brown glass vials at 4 °C until use. Since the actual exposure concentrations were close to the nominal ones [23,24], the nominal concentrations were used for the results presented throughout this study. The control and treatment groups were fed spirulina powder one time per day for the experimental period and all experiments were performed at 10 ± 1 °C.

#### 2.2.3. Sampling of Scallop

After exposure, six scallops were sampled from each group at 1, 3, 5, 10, 15, 20, 25, and 30 d. At each time point, the adductor muscle, mantle, disgestive gland, gills, and gonads were sampled. All samples were stored at −20 °C until analysis.

### 2.3. Sample Preparation

#### 2.3.1. Determination of Semicarbazide

The analysis of free and bound semicarbazide in total was performed following Announcement No. 783 of the Ministry of Agriculture-1-2006 with some modifications.

In brief, 2.0 g homogenized tissue was weighed in 50 mL polypropylene centrifuge tubes. 50 μL internal standard of semicarbazide-^13^C,^15^N_2_ (100 ng/mL) was added and mixed with 5 mL 0.2 M HCL and 150 μL 2-nitrobenzaldehyde (0.05 M in dimethyl sulfoxide, DMSO). The mixture was incubated in an air bath at 37 °C for 16 h.

After incubation, the pH of the solution was adjusted to 7.0~7.5 by addition of 1 M dipotassium hydrogen orthophosphate. Then 8 mL ethyl acetate was added into the tube, mixed well and centrifuged at 8000 rpm for 5 min, whereupon the supernatant was decanted and the pellet discarded. The extracts were concentrated to dryness under a nitrogen stream at 45 ℃. The residue was dissolved in methanol: water (5:95). The solutions were ultrasonicated for 1 min and centrifuged at 14,000 rpm for 10 min. The supernatants were filtered through 0.22 μm syringe filters.

#### 2.3.2. Determination of Tissue-Bound Semicarbazide

Free semicarbazide was released by washing the tissues with 6 mL of methanol/water (50:50; *v*/*v*) followed by ultrasonic extraction for 5 min and centrifugation at 6000 rpm for 5 min. The supernatant was discarded and the sample was washed with 6 mL methanol/water (75:25; *v*/*v*), 6 mL of methanol and 6 mL of water in succession. The supernatant was discarded between each washing step. The washed sample was then treated as described for total semicarbazide determination.

### 2.4. Instruments and Chromatographic Conditions

Semicarbazide was analyzed using a AB Sciex 5500 Qtrap system equipped with an ESI source and interfaced to a LC 20AD HPLC system (Shimadzu, Kyoto, Japan). Data were acquired and analyzed using Analyst Software 2.0.

Mass spectrometry data were collected in positive ion mode with the following LC-MS/MS parameters: ion spray voltage (IS), 5500 V; ion source temperature (TEM), 550 °C; collision activated dissociation (CAD), medium; curtain gas (CUR), 30 psi; nebulizing gas (Gas 1), 35 psi; heater gas (Gas 2), 35 psi; declustering potential (DP), 80 V; entrance potential (EP): 10 V; collision cell exit potential (CXP), 10 V.

Multiple reaction monitoring (MRM) mode was applied for analytes quantification. The optimized parameters for analytes and the internal standard were as follows. The quantitative ion pairs were *m*/*z* 209/166 for semicarbazide, 212/168 for semicarbazide-^13^C,^15^N_2_ and the qualitative ion pair were *m*/*z* 212/192 for semicarbazide.

Chromatographic separation was performed on a Kinetex XB C_18_ (2.6 μm, 2.1 × 100 mm) (Phenomenex, Torrance, CA, USA). Gradient elution used the mobile phases (A) 2 mM ammonium acetate as aqueous solution and (B) methanol at a flow rate of 300 μL min^−1^ as follows: 0–0.5 min, 10% B; 0.5–4.0 min, 10–95% B; 4.0–5.5 min, 95%; 5.5–6.0 min, 95–10% B; 6.0–7.0 min 10% B. The valve was set to direct LC flow to the mass spectrometer from 1.5 to 5.5 min with the remaining LC eluent diverted to waste. Identification and quantification of the analytes were based on MRM.

### 2.5. Calculation of the Bioconcentration Factor

Scallops were farmed in the aquarium, so the sediment influence was ignored. The bioconcentration factor (BCF) was defined as the ratio between the semicarbazide concentrations in scallops and seawater. The computational formula was as follows.

BCF = C_biomass_/C_water_

where C_biomass_ and C_water_ are the sample concentrations in scallops and its corresponding seawater.

### 2.6. Statistical Analysis

Significant differences for the correlation coefficient R were analyzed by consulting the correlation coefficient significance test table [40]. The data were presented as the Means ± SD (*n* = 6). Nonparametric Mann–Whitney U test was used to analyze the difference of tissue-bound semicarbazide proportion between groups, a Kruskal–Wallis test, and two-way ANOVA were carried out in order to assess significant differences in the concentrations of semcarbazide between tissues and time, and Spearman correlation test was used to analyze the correlation. The data analysis was performed using the Statistical Package for the Social Sciences (SPSS 18.0).

## 3. Results

### 3.1. Exposure of Scallops to Semicarbazide in Aquaculture Seawater

**Figure 1:**
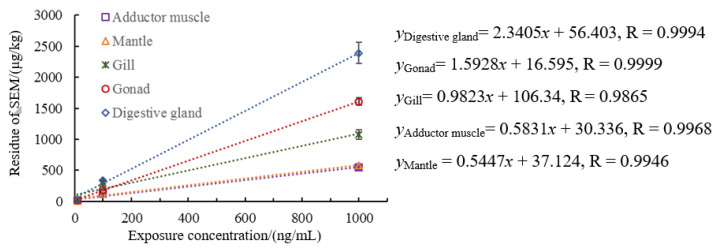
Relationship between residual semicarbazide in scallops and exposure concentration.

We detected semicarbazide in the adductor muscle, mantle, gills, disgestive gland, and gonads of scallops exposed to seawater contaminated by semicarbazide. In this method, the limits of detection (LOD) of semicarbazide was 0.25 μg/kg, while the limits of quantitation (LOQ) was 0.5 μg/kg, and the concentration values obtained from the experiment were higher than LOD. According to the literature, the semicarbazide levels near the Chaohe River estuary, in western Laizhou Bay and in Jincheng and Sishili Bay were 0.18–70.6 μg/kg, 10^−11^ kg/L and 0.011–0.093 μg/L, respectively [28,29,30]. Based on the pollution status, we set three exposure concentrations of low, medium and high. We found a good linear relationship between semicarbazide level in scallop tissues and the exposure concentration. The correlation coefficients for the linear equations for each tissue were all >0.917 and very significant (Figure 1) (*p* < 0.01). These data indicated that an increase of semicarbazide exposure concentration in seawater resulted in corresponding significant positive increases in the tissues. The Kruskal–Wallis test revealed that there were significant differences among tissues (Kruskal–Wallis chi-squared = 26.998, df = 4, *p* < 0.01), suggesting that the concentrations of semcarbazide was dependent on the tissues.

### 3.2. Morphology of Semicarbazide in Scallop

**Figure 2:**
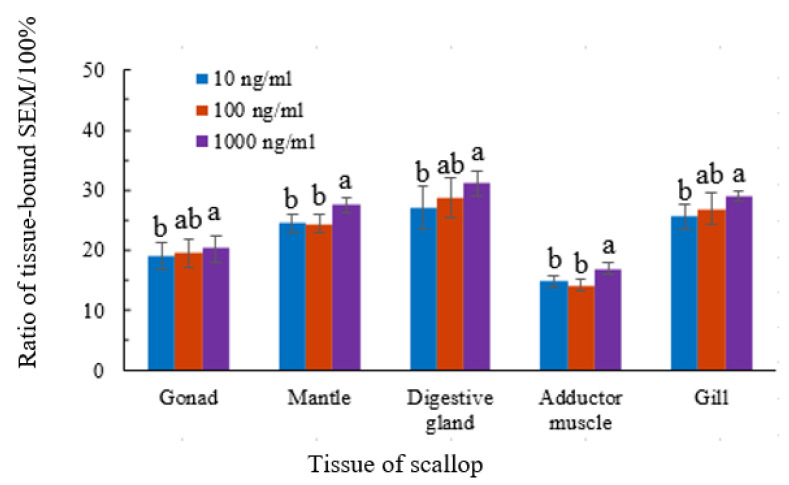
Proportion of tissue-bound semicarbazide in different scallop tissues. a,b mean values with different letters on vertical bars mean significant difference (*p* < 0.05).

We determined the amounts of total and tissue-bound semicarbazide in the scallops and the free form was present in all tissues. The proportion of tissue-bound semicarbazide ranged from 12.1 to 32.7%. Nonparametric Mann–Whitney U test revealed that, for the tissue of gonad, digestive gland, and gill, the proportion of tissue-bound semicarbazide had significant difference between 10 ng/mL group and 1000 ng/mL group (*p* < 0.05), but no significant difference between 10 ng/mL group and 100 ng/mL, or 100 ng/mL and 1000 ng/mL (*p* > 0.05). For the tissue of mantle and adductor muscle, the proportion of tissue-bound semicarbazide had significant difference between 1000 ng/mL group and 100 ng/mL or 10 ng/mL (*p* < 0.05), but no significant difference between 10 ng/mL group and 100 ng/mL (*p* > 0.05). The proportion of tissue-bound semicarbazide remained relatively stable and did not increase with the increase of exposure concentration. The ratio of tissue-bound semicarbazide in gonads was 16.9–22.4%, mantle 22.9–28.5%, disgestive gland 27.3–32.7%, adductor muscle 12.1–17.8%, and gills 23.1–30.7%. The proportion of tissue-bound semicarbazide was the highest in disgestive gland tissue and lowest in adductor muscle. The proportion of tissue-bound semicarbazide in different tissues from high to low was as follows: viscera mass > gill > mantle > gonad > adductor muscle (Figure 2).

### 3.3. Semicarbazide Ci-Ti

**Figure 3:**
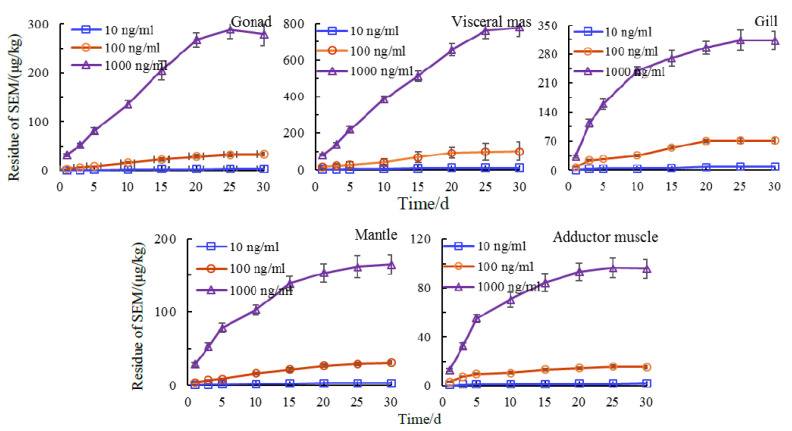
*Ci*-*Ti* of tissue-bound semicarbazide residue in scallop with exposure time.

We then examined accumulation of semicarbazide from culture water at 10, 100, and 1000 ng/mL over time. Drug concentration–time curve (*Ci*-*Ti*) refers to the curve of drug concentration changing with time, which reflects the dynamic process of drug in vivo to a certain extent. In the early stage of exposure, the tissue-bound semicarbazide increased rapidly and stabilized at increasing concentrations. The stable accumulation time for accumulation in gonads, adductor muscle and gills was 25 d while a small increasing trend was seen for bound semicarbazide in viscera and mantle. The steady-state accumulation time of semicarbazide in scallop tissues required an extended period of time and residual semicarbazide in the same tissues also differed. Two-ways ANOVA was used to analyze the effects of exposure concentration and exposure time on the residues of bound SEM in scallop. The results showed that there was no interaction between exposure concentration and exposure time on the residues of tissue-bound SEM in scallop. For the same tissue, the effects of exposure concentration and exposure time on tissue-bound SEM residue were significant (*p* < 0.001), which means an increase of exposure concentration or exposure time resulted in corresponding tissue-bound SEM increases in levels. However, the enrichment rate decreased after 20 d and gradually tended to be stable (Figure 3).

### 3.4. Tissue Distribution of Semicarbazide

**Figure 4:**
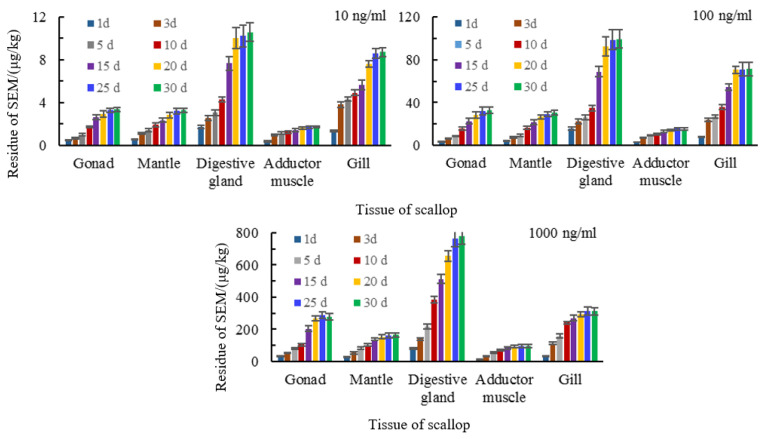
Distribution of tissue-bound semicarbazide in scallop tissues.

An increase of exposure concentration of semicarbazide resulted in different levels in the tissues we examined. The highest concentrations of tissue-bound semicarbazide were found in the disgestive gland and the gills while concentrations in gonads and mantle were similar and the adductor muscle showed the lowest level. The levels of tissue-bound semicarbazide in disgestive gland were 6–8 times of adductor muscle, in gills were 3–5 times, and in gonads and mantle were 2–3 times (Figure 4). The liver and gills are the primary tissues involved in drug metabolism and they were also the tissues with the highest tissue-bound semicarbazide levels. The total concentration of semicarbazide in scallops could be ranked as viscera > gill > gonad > mantle > adductor muscle. In contrast, the total semicarbazide level was greatest for gonads > mantle.

### 3.5. Bioaccumulation of Semicarbazide in Scallop

**Figure 5:**
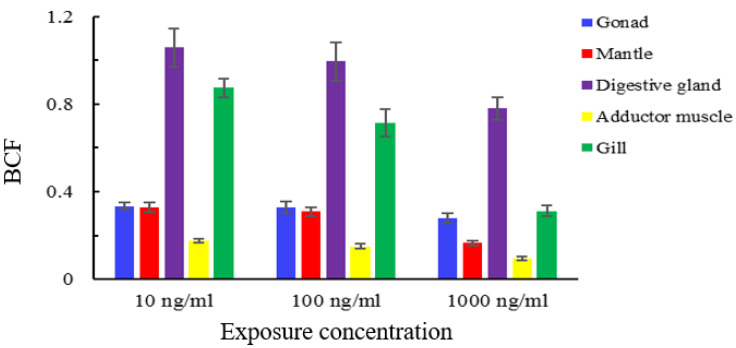
BCF of tissue-bound semicarbazide at 30 days in scallop.

Bioaccumulation refers to the ability of pollutants to enter and accumulate in organisms from the environment and then be transferred and accumulate in the food chain. Higher levels of bioaccumulation pose higher chronic harm to organisms across the food chain [41,42,43]. The BCF is used to measure the accumulation trend of pollutants in organisms and to describe the bioaccumulation effect of pollutants. The BCF of semicarbazide in the same tissue gradually increased and tended to be stable with the increase of time at each exposure concentration. When the pollutants enter the scallop, the process of accumulation and metabolism coexist. For instance, in the early stage, the accumulation rate was higher than the metabolic rate and in the later stages the accumulation and metabolic processes gradually reached a balance. However, different tissues had different bioaccumulation ability for semicarbazide. At 30 days, the BCF of tissue-bound semicarbazide in disgestive gland had the highest bioaccumulation ability while the muscle had the lowest at the same exposure concentration. The order for the BCF was viscera mass > gill > gonad > mantle > adductor muscle (Figure 5).

**Figure 6:**
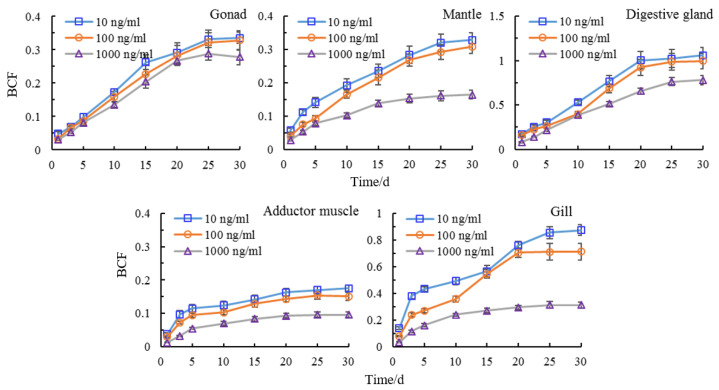
BCF of tissue-bound semicarbazide in scallop.

The BCF of semicarbazide in the same tissues differed between exposure concentrations. The semicarbazide increased with the increase of exposure concentration but the BCF was not proportional to this. For the same tissue, the BCF was the highest at 10, 100, and 1000 ng/mL in decreasing order. The same rule was observed in adductor muscle, mantle, gonad, gill, and viscera mass. Spearman correlation test was used to analyze the correlation between exposure concentration and BCF. The results showed that there was a significant negative correlation (*p* < 0.01) which indicating that the BCF of semicarbazide in scallops exposed at low concentrations was greater than that at high concentration (Figure 6).

## 4. Discussion

The European Commission has suggested a minimum required performance level of 1 μg kg^−1^ for the analysis of nitrofurans (Commission Decision 2003/181/EC) [44]. The determination limit value of nitrofuran metabolites is 1.0 μg/kg for aquatic product quality and safety monitoring (risk monitoring) (The Ministry of Agriculture and Rural Affairs of Agro-product Safety and Quality Department Documents [2020] No. 1) and in the veterinary drug residue monitoring plan (The Ministry of Agriculture and Rural Affairs of Fisheries Bureau [2020] No. 4) in China. The semicarbazide content in shellfish near the polluted Chaohe River ranged from 3.14 to 6.46 μg/kg and exceeded the food safety limit of 1 μg/kg proposed by European Union [28]. In our study, semicarbazide in adductor muscles of scallops could reach 2.19 μg/kg after exposure of semicarbazide in seawater for 1 d at 10 ng/mL. The semicarbazide levels in the marine environment and shellfish in Sishili Bay were positively correlated with the content in shellfish and in the seawater [45]. Semicarbazide content in shellfish has also been linked to increases in seawater for Laizhou Bay [29] and those results were similar to our study. This indicated that when aquaculture water was polluted by low concentrations of semicarbazide, there was a risk of accumulation in shellfish tissues.

The form of semicarbazide in the shrimp *Macrobrachium rosenbergii* was examined by exposure to culture water containing 50 mg/L nitrofurazone for one week. The results indicated that the free and bound semicarbazides had increased significantly. The bound semicarbazides in the muscle tissue were about one-quarter of the total while the bound semicarbazide in the shell accounted for >65% of the total [37]. An analytical method for the determination of bound residues of nitrofuran drugs developed by Chu found that 95% of the unbound semicarbazides could be removed in the prewashing steps (washed with 50% aqueous MeOH, EtOAc, and EtOH, respectively) [36]. Semicarbazide in *Macrobrachium nipponense* mainly existed in the free form in muscle and disgestive gland with proportions of free semicarbazide at 67.35% and 72.4%, respectively [46]. In the shell, eyestalks, pereopod, cephalothorax, and gills semicarbazide primarily existed in the tissue-bound form with proportion of 89.50%, 87.16%, 85.68%, 80.48%, and 73.30%, respectively.

The ratio of semicarbazide in the scallops used in our study was similar to that found in muscle tissue of the shrimp *Macrobrachium rosenbergii* treated with nitrofurazone, although the absolute levels differed, indicating species-specific differences. However, semicarbazide in scallops originated from free semicarbazide in the environment while the study using shrimp was metabolized via nitrofurazone [37]. The metabolic pathways also differ between semicarbazide and nitrofurazone. In our study, although the drug was not administered via nitrofurazone, it primarily existed in free form in the animals. The proportion of bound semicarbazide was only about 10–30%. A previous study indicated that nitrofuran antibiotics after storage and cooking were stable, and between 67% and 100% of the residue remained, respectively [35]. This demonstrated that these metabolites are largely resistant to conventional cooking techniques and would continue to pose a health risk even after consumer processing. Although the ratio of tissue-bound semicarbazide was low for scallops, the risks to human health through the tissue-bound semicarbazide may not be low due to its stability.

An extension of exposure time or a concentration increase was directly proportional to accumulation in scallop tissues. However, the scallops accumulated semicarbazide up to an equilibrium point. The BCF decreased with increased concentration and is most likely related to metabolic functions. Exposure to high concentrations of semicarbazide would result in longer excretion times. In order to avoid injury, the body responds by self-regulation and aquatic animals can significantly improve their antioxidant enzyme activities to reduce the damage caused by pollutants and their metabolites [47].

Environmental pollutants enter the food, sediment and water and affect the quality and safety of aquaculture products. For example, when furazolidone was fed continuously for 12 days at 0.01% therapeutic dose to chickens, AOZ residuals in liver and muscle were 1.1 and 0.33 μg/kg, respectively, indicating that nitrofuran metabolites can originate in diet and environmental pollution [48]. The use of feed or feed raw materials contaminated by antibiotics can lead to their accumulation in shrimp [49]. An assessment of nitrofurans, nitroimidazoles and tetracyclines in animal feed indicated a significant probability of antibiotic contamination [50]. An exposure assessment of the Irish population to nitrofuran metabolites from different food commodities in 2009–2010 indicated that semicarbazide was the contaminant most frequently identified (range, 0.09–1.27 μg kg^−1^) and both adults, teenagers, and children had been exposed to one or more of the foods containing semicarbazide [51]. Although these levels were below EFSA-estimated safe levels, there were still risks to human health because of the newly discovered toxic effects of semicarbazide.

## 5. Conclusions

In this study, the scallop *Chlamys farreri* was selected as the research object. When it was exposed to the environment polluted by semicarbazide, the forms and residue of semicarbazide in scallop were determined. We found that pollution of the culture environment by semicarbazide led to accumulation in the animals even with low pollution levels. Endogenous semicarbazides have been found in marine food (such as seaweed and shrimp) and the relationship between endogenous semicarbazides and environmental semicarbazides needs further study. The newly discovered toxic effects of semicarbazide indicate that it is necessary to monitor environmental semicarbazide levels and residues in cultured shellfish to ensure food quality and safety. The determination of tissue-bound semicarbazide is more meaningful when evaluating semicarbazide hazards.

## Data Availability

The datasets collected and analyzed during the current study are available from the corresponding author upon request.

## References

[1] EunChae R., Ji-Sung P., Sib Sankar G., Se Chang P. (2020). A simplified modification to rapidly determine the residues of nitrofurans and their metabolites in aquatic animals by HPLC triple quadrupole mass spectrometry. Environ. Sci. Pollut. Res..

[2] Bogialli S., Di Corcia A. (2009). Recent applications of liquid chromatography–mass spectrometry to residue analysis of antimicrobials in food of animal origin. Anal. Bioanal. Chem..

[3] Wu W., Yang S., Liu J., Mi J., Dou L., Pan Y., Mari G.M., Wang Z. (2020). Progress in immunoassays for nitrofurans detection food and agricultural immunology. Food Agric. Immunol..

[4] Wang K., Kou Y., Wang M., Ma X., Wang J. (2020). Determination of nitrofuran metabolites in fish by ultraperformance liquid chromatography-photodiode array detection with thermostatic ultrasound-assisted derivatization. ACS Omega.

[5] Ralica K. (2020). Antibiotic residues and human health hazard–review. Bulg. J. Agric. Sci..

[6] (1995). Commission Regulation (EC) No 1442/95. Amending annexes I, II, III and IV of Council Regulation (EEC) No 2377/90 laying down a community procedure for the establishment of maximum residue limits of veterinary medicinal products in foodstuffs of animal origin. Off. J. Eur. Communities.

[7] FDA (2002). Topical nitrofurans; extralabel animal drug use; order of prohibition. Fed. Regist. Dep. Heal. Hum. Serv..

[8] Ministry of Agriculture of China (2002). Announcement No.235 of the Ministry of Agriculture of China, Maximum Residue Limits of Veterinary Drugs in Animal Food.

[9] Yu W.L., Liu W.H., Tian W.R., Li X.T., Wang X.H. (2019). Semicarbazide universality study and its speculated formation pathway. J. Food Saf..

[10] Hron R., Jursic B.S. (2014). Preparation of substituted semicarbazides from corresponding amines and hydrazines via phenyl carbamates. Tetrahedron Lett..

[11] Subashchandrabose S., Babu N.R., Saleem H., Padusha M.S.A. (2015). Vibrational studies on (E)-1-((pyridine-2-yl) methylene) semicarbazide using experimental and theoretical method. J. Mol. Struct..

[12] Adam B., Benjamin P.Y.L., David L., Stephen W.S. (2004). Semicarbazide formation in azodicarbonamide-treated flour: A model study. J. Agric. Food Chem..

[13] Chen L., Cui H., Dong Y.L., Guo D.Q., He Y.J., Li X.J., Yuan Z.B., Zou H. (2016). Simultaneous detection of azodicarbonamide and the metabolic product semicarbazide in flour by capillary electrophoresis. Food Anal. Method.

[14] Wei T.F., Li G.K., Zhang Z.M. (2017). Rapid determination of trace semicarbazide in flour products by high-performance liquid chromatography based on a nucleophilic substitution reaction. J. Sep. Sci..

[15] Hoenicke K., Gatermann R., Hartig L., Mandix M., Otte S. (2004). Formation of semicarbazide (SEM) in food by hypochlorite treatment: Is SEM a specific marker for nitrofurazone abuse?. Food Addit. Contam..

[16] Bendall J.G. (2009). Semicarbazide is non-specific as a marker metabolite to reveal nitrofurazone abuse as it can form under Hofmann conditions. Food Addit. Contam..

[17] Vlastos D., Moshou H., Epeoglou K. (2009). Evaluation of genotoxic effects of semicarbazide on cultured human lymphocytes and rat bone marrow. Food Chem. Toxicol..

[18] Richard H.S., Ludovica V., Walburga S., Lucie R. (2015). Why semicarbazide (SEM) is not an appropriate marker for the usage of nitrofurazone on agricultural animals. Food Addit. Contam. A.

[19] Kwon J.W. (2017). Semicarbazide: Natural occurrence and uncertain evidence of its formation from food processing. Food Control..

[20] Magyar K., Mészáros Z., Mátyus P. (2001). Semicarbazide-sensitive amine oxidase. Its physiological significance. Pure Appl. Chem..

[21] Dawson D.A., Rinaldi A.C., Pöch G. (2002). Biochemical and toxicological evaluation of agent-cofactor reactivity as a mechanism of action of osteolathyrism. Toxicology.

[22] Macedo C.E., Martinez R.C., Albrechet-Souza L., Molina V.A., Brandão M.L. (2007). 5-HT_2_- and D_1_-mechanisms of the basolateral nucleus of the amygdala enhance conditioned fear and impair unconditioned fear. Appl. Behav. Brain Res..

[23] Gao S., Wang W., Tian H., Zhang X.N., Guo L.L., Ru S.G. (2014). An emerging water contaminant, semicarbazide, exerts an anti-estrogenic effect in zebrafish (*Danio rerio*). Bull. Env. Contam. Toxicol..

[24] Yu M., Feng Y.L., Zhang X.N., Wang J., Tian H., Wang W., Ru S.G. (2017). Semicarbazide disturbs the reproductive system of male zebrafish (*Danio rerio*) through the GABAergic system. Reprod. Toxicol..

[25] Yue Z.H., Yu M., Zhao X.N., Wang J., Ru S.G. (2018). The anti-androgenic effect of chronic exposure to semicarbazide on male Japanese flounder (*Paralichthys olivaceus*) and its potential mechanisms. Comp. BioChem. Phys. C.

[26] Yue Z.H., Yu M., Zhao H.F., Wang J., Zhang X.N., Tian H., Wang W., Ru S.G. (2018). The anti-estrogenicity of chronic exposure to semicarbazide in female Japanese flounders (*Paralichthys olivaceus*), and its potential mechanisms. Mar. Pollut. Bull..

[27] Tian X.H., Xu Y.J., Zheng H.W., Cui Y.M., Jiang F., Gong X.H. (2020). Semicarbazide exposure induces histological damage and enzymatic reactions in *apostichopus japonicas*. Mod. Food Sci. Technol..

[28] Xu Y.J., Sun X.K., Tian X.H., Gong X.H., Zhang X.Z., Zhang L.M. (2010). Survey of semicarbazide contamination in castal waters adjacent to the chaohe river estuary. Oceanol. Limnol. Sin..

[29] Tian X.H., Xu Y.J., Song X.K., Gong X.H., Liu Y.H., Zhou Q.L., Wang Z.Q., Xia C.H. (2016). Temporal and spatial distribution of semicarbazide in western Laizhou Bay. Mar. Pollut. Bull..

[30] Tian X.H., Xu Y.J., Gong X.H., Han D.F., Wang Z.Q., Zhou Q.L., Sun C.X., Ren C.B., Xue J.L., Xia C.H. (2017). Environmental status and early warning value of the pollutant Semicarbazide in Jincheng and Sishili Bays, Shandong Peninsula, China. Sci. Total Env..

[31] Nouws J.F., Laurensen J. (1990). Postmortal degradation of furazolidone and furaltadone in edible tissues of calves. Vet. Q.

[32] Kim D., Kim B., Hyung S.W., Lee C.H., Kim J. (2015). An optimized method for the accurate determination of nitrofurans in chicken meat using isotope dilution–liquid chromatography/mass spectrometry. J. Food Compos. Anal..

[33] Rizzo G., Baroni L. (2018). Soy, soy foods and their role in vegetarian diets. Nutrients.

[34] Yuan G.X., Zhu Z., Yang P., Lu S.Y., Liu H.H., Liu W.J., Liu G.H. (2020). Simultaneous determination of eight nitrofuran residues in shellfish and fish using ultra-high performance liquid chromatography–tandem mass spectrometry. J. Food Compos. Anal..

[35] Cooper K.M., Kennedy D.G. (2007). Stability studies of the metabolites of nitrofuran antibiotics during storage and cooking. Food Addit. Contam..

[36] Chu P.S., Lopez M.I. (2005). Liquid chromatography-tandem mass spectrometry for the determination of protein-bound residues in shrimp dosed with nitrofurans. J. Agric. Food Chem..

[37] Van Poucke C., Detavernier C., Wille M., Kwakman J., Sorgeloos P., Van Peteghem C. (2011). Investigation into the possible natural occurence of semicarbazide in *Macrobrachium rosenbergii* prawns. Agr. Food Chem..

[38] FAO (2020). The State of World Fisheries and Aquaculture 2020.

[39] Bureau of Fisheries of Ministry of Agriculture and Rural Affairs of the PRC, National Fisheries Technology Extension Center, China Society of Fisheries (2020). Part Two: Production. 2020 China Fishery Statistical Yearbook, 1st ed.

[40] Department of probability and statistics, Institute of mathematics, Chinese Academy of Sciences (1979). The critical value (*r*_a_) table of correlation coefficient ρ = 0. Common Mathematical Statistics.

[41] Zenker A., Cicero M.R., Prestinaci F., Bottoni P., Carere M. (2014). Bioaccumulation and biomagnification potential of pharmaceuticals with a focus to the aquatic environment. J. Environ. Manag..

[42] Cammilleri G., Galluzzo F.G., Fazio F., Pulvirenti A., Vella A., Dico G.M.L., Macaluso A., Ciaccio G., Ferrantelli V. (2019). Mercury detection in benthic and pelagic fish collected from western Sicily (Southern Italy). Animals.

[43] Giangrosso G., Cammilleri G., Macaluso A., Vella A., D’Orazio N., Graci S., Dico G.M.L., Galvano F., Giangrosso M., Ferrantelli V. (2016). Hair mercury levels detection in fishermen from Sicily (Italy) by ICP-MS method after microwave-assisted. Bioinorg Chem. Appl..

[44] (2003). Commission Decision (2003/181/EC). Amending decision 2002/657/EC as regards the setting of minimum required performance limits (MRPLs) for certain residues in food of animal origin. Off. J. Eur. Union.

[45] Yu Z.Q., Xu Y.J., Tian X.H., Liu Y.H., Song X.K., Ren C.B., Huang H., Liu Y., Zhang L.M. (2013). Semicarbazide bioaccumulation in seashells of Sishili Bay. Mar. Environ. Sci..

[46] Shu X.J., Cheng B., Xu J.J., Xiong Y.W., Song W., Song Y., Han G., Jiang S.F. (2020). Study on the characteristics of semicarbazide (SEM) in *Macrobrachium nipponense* during the culture period. Freshw. Fish..

[47] Pan L.Q., Ren J.Y., Liu J. (2006). Responsed of antioxidant systems and LPO level to benzo(a) pyrenye and benzo(k) fluoranthene in the haemolymph of the scallop *Chlamys farreri*. Environ. Pollut..

[48] Mccracken R.J., Van Rhijn J.A., Kennedy D.G. (2005). The occurrence of nitrofuran metabolites in the tissues of chickens exposed to very low dietary concentrations of the nitrofurans. Food Addit. Contam..

[49] Jakiul Islam M., Liza A.A., Mohsinul Reza A.H.M., Shaheed Reza M., Khan M.N.A., Kamal M. (2014). Source identification and entry pathways of banned antibiotics nitrofuran and chloramphenicol in shrimp value chain of Bangladesh. Eurasia J. BioSci..

[50] Rizal G.M., Gyeltshen J., Gyeltshen K. (2018). Evaluation of animal feeds for the presence of three important antibiotic classes in Bhutan. J. Glob. Antimicrob. Resist..

[51] Radovnikovic A., Conroy E.R., Gibney M., O’Mahony J., Danaher M. (2013). Residue analyses and exposure assessment of the Irish population to nitrofuran metabolites from different food commodities in 2009–2010. Food Addit. Contam. A.

